# A, B, C’s of Trk Receptors and Their Ligands in Ocular Repair

**DOI:** 10.3390/ijms232214069

**Published:** 2022-11-15

**Authors:** Akash Gupta, Jeremias G. Galletti, Zhiyuan Yu, Kevin Burgess, Cintia S. de Paiva

**Affiliations:** 1 Ocular Surface Center, Cullen Eye Institute, Department of Ophthalmology, Baylor College of Medicine, Houston, TX 77030, USA; 2 Institute of Experimental Medicine, Vuelta de Obligado 2490, Buenos Aires C1428 ADN, Argentina; 3 Department of Chemistry, Texas A&M University, College Station, TX 77842, USA

**Keywords:** neurotrophins, nerve growth factor, Trk receptors, ocular surface, neurotrophic keratitis

## Abstract

Neurotrophins are a family of closely related secreted proteins that promote differentiation, development, and survival of neurons, which include nerve growth factor (NGF), brain-derived neurotrophic factor, neurotrophin-3, and neurotrophin-4. All neurotrophins signal through tropomyosin receptor kinases (TrkA, TrkB, and TrkC) which are more selective to NGF, brain-derived neurotrophic factor, and neurotrophin-3, respectively. NGF is the most studied neurotrophin in the ocular surface and a human recombinant NGF has reached clinics, having been approved to treat neurotrophic keratitis. Brain-derived neurotrophic factor, neurotrophin-3, and neurotrophin-4 are less studied neurotrophins in the ocular surface, even though brain-derived neurotrophic factor is well characterized in glaucoma, retina, and neuroscience. Recently, neurotrophin analogs with panTrk activity and TrkC selectivity have shown promise as novel drugs for treating dry eye disease. In this review, we discuss the biology of the neurotrophin family, its role in corneal homeostasis, and its use in treating ocular surface diseases. There is an unmet need to investigate parenteral neurotrophins and its analogs that activate TrkB and TrkC selectively.

## 1. Introduction

Neurotrophins are closely related secreted proteins that promote differentiation, development, and survival of neurons, which include nerve growth factor (NGF), brain-derived neurotrophic factor, neurotrophin-3, and neurotrophin-4. Neurotrophins were first described by Nobel laureate Rita Levi-Montalcini more than 60 years ago and since then have been associated with profound impacts on cell development and survival in the nervous system [1]. NGF was first implicated when scientists were searching for survival factors which maintained a balance between organ size and magnitude of innervation [2]. The purpose of this review is to describe the role of neurotrophins and their receptors in corneal homeostasis, neuron survival, and wound healing. In addition, the molecular structure of neurotrophins and the signaling pathways through which neurotrophins exert their effects are elucidated. Lastly, in this review, we provide an essential summary of the evolution of clinical trials that have led to the utilization of neurotrophins, in particular, NGF in ocular surface disease treatment, and we present an up-to-date discussion on current developments including neurotrophin analogs. A list of abbreviations used in this review can be found in Table 1.

The nervous system widely expresses tropomyosin receptor kinase (Trk) receptors, of which TrkA–C are selective (but not specific) for the neurotrophins NGF, brain-derived neurotrophic factor (and neurotrophin-4), and neurotrophin-3, respectively [1]. For instance, neurotrophin-3 binds TrkA, but with lower affinity than for TrkC [3,4]. All neurotrophins also bind “the death receptor” p75 which can promote apoptosis, survival, or otherwise regulate Trk activities [5,6,7,8]. Expression of p75 can also determine whether neurotrophin-3 binds and activates TrkA [9,10].

Evolutionarily, Trk receptors are believed to have originated at least 500 million years ago, expanding plasticities in nervous systems of vertebrates [1]. The linkage between the neurotrophins and Trk families is thought to have led to their co-evolution, hence, the specific and varied functions neurons possess [11]. Four neurotrophins of mammals (NGF, brain-derived neurotrophic factor, neurotrophin-3, and neurotrophin-4) originated from the same ancestral gene [11]. Neurotrophin genes are found on chromosomes 1p for NGF, 11p for brain-derived neurotrophic factor, 12p for neurotrophin-3, and 19q for neurotrophin-4 [11]. NGF is made and secreted by target organs after which it is drawn into nerve terminals by receptor-mediated endocytosis and shuttled along axons to cell bodies where it promotes neuronal growth [12]. Other neurotrophin sources include NGF from Schwann cells and fibroblasts after peripheral nerve injury [12], expression of neurotrophins during development in areas that sensory axons pass through to get to their endpoint [13], and generation of neurotrophins by neurons [14].

## 2. Molecular Structure

**Figure 1:**
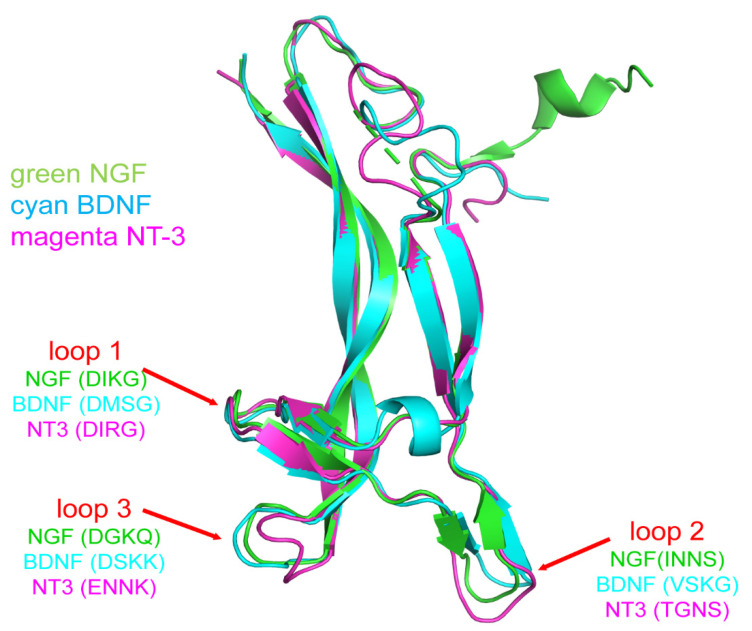
Structures of NGF, brain-derived neurotrophic factor (BDNF), and neurotrophin-3 (NT-3) from crystallography. Abbreviations inside parenthesis are the standard one letter code denotations for amino acids in the loops.

Neurotrophins are homodimeric with about 50% sequence homology [15] (Figure 1). NGF has an extended shape with the middle portion comprising two pairs of beta strands running antiparallel [15]. One end has three hairpin loops, and on the other end, there is a cysteine knot motif from three disulfide bonds, where two of the bonds form a closed ring through which the third disulfide bridge passes [15]; this Cys knot maintains the protein folding [15]. Two NGF monomers form homodimers [15]. Brain-derived neurotrophic factor, neurotrophin-3, neurotrophin-4, and other dimeric growth factors such as platelet-derived growth factor, human chorionic gonadotropin, and transforming growth factor-beta have similar structures. The differentiating factor for neurotrophins is their parallel dimerization mechanism endowing them with dumbbell shapes [15].

## 3. TrK and Signaling Pathways (TrkA–C)

Neurotrophin receptors TrkA–C have similar extracellular domains that are densely populated with cysteine, leucine repeats, and two immunoglobulin-like folds. An intracellular tyrosine kinase region responds to conformational changes in the extracellular domain via a helix that crosses the membrane. Of the extracellular portions, the immunoglobulin C2-like domains (domains 4 and 5) have been shown in multiple studies to have a direct role in binding with the neurotrophin ligand. In vitro, domain 4 is needed for effective folding of ligand binding domain 5 [16]. The immunoglobulin-like domains prevent unregulated auto-activation of the Trk receptors and facilitate selective binding with NGF, brain-derived neurotrophic factor, neurotrophin-3, and neurotrophin-4 [1].

**Figure 2:**
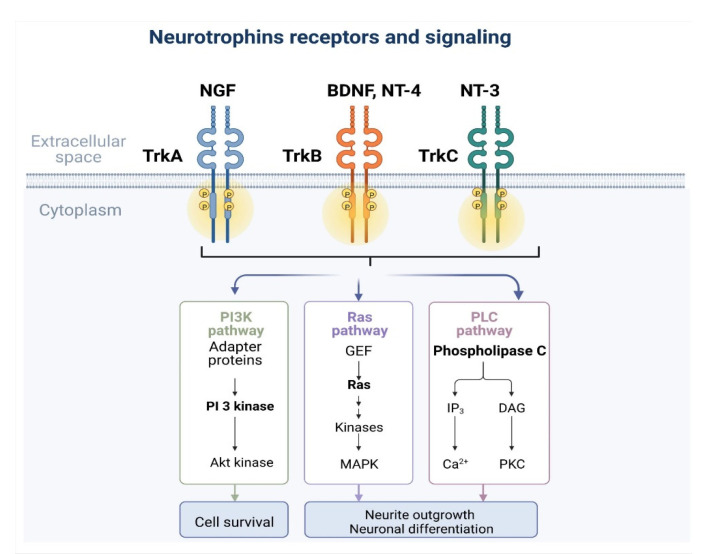
Schematic of Trk receptors and preference binding for neurotrophins. p75 signaling not depicted. Image created using Biorender.

Signaling from Trk receptors is initiated by binding neurotrophin ligands, predominantly those most closely associated with each receptor (Figure 2). TrkA is activated by NGF, TrkB is activated by brain-derived neurotrophic factor and neurotrophin-4, and TrkC is activated by neurotrophin-3 [17]. The p75 neurotrophin receptor enhances the specificity of each neurotrophin for its respective receptor [18].

The neurotrophin•Trk complexes activate tyrosine autophosphorylation in the cytoplasmic Trk receptor region leading to tyrosine kinase activity [18], creating protein attachment sites. Attachment of those proteins initiates signaling via the phosphatidylinositol-3-kinase proteinase B (also known as Akt), Ras/Raf/Erk (rat sarcoma virus/rapidly accelerated fibrosarcoma/extracellular signal related kinase), phospholipase C-γ-Ca^2+^, NFκB, and atypical protein kinase C pathways [18].

The phosphatidylinositol-3-kinase pathway begins with phosphatidylinositol-3-kinase creating phosphatidylinositides which activate phosphatidylinositide-dependent protein kinase [18]. Combined, these factors activate protein kinase Akt which negatively regulates proteins in the apoptotic pathway such as Bcl-2 antagonist of cell death (Bad), glycogen synthase kinase 3-β, IκB, and forkhead transcription factor. Protein kinase B promotes neuronal cell survival through this mechanism [18,19].

Ras/MAPK/Erk (Ras/extracellular signal related kinase) activation is also important for neurotrophin-mediated neuronal growth. MAP kinase signaling begins with protein phosphorylation of the adaptor protein Src homology and collagen (Shc), which recruits growth factor receptor-bound protein 2 (Grb2) and, in combination with son of sevenless protein (SOS), activates Ras [18] through phosphorylation and promotes Erk signaling [20].

Phospholipase C-γ-Ca^2+^ involves activation of phospholipase C-γ-1 which generates inositol trisphosphate and diacylgylerol through cleavage [18]. Inositol trisphosphate causes release of calcium stores that activate enzymes such as protein kinase C, and protein kinase C-delta δ, and ultimately promote neuronal growth and activation of extracellular signal-regulated kinase [21].

Ocular surfaces and lacrimal glands express TrkA–C [22,23,24,25,26,27]. Elevated levels of neurotrophins are found in tears and serum of dry eye patients [28,29] and after ocular injury [30,31,32]. In the next section, we describe NGF, brain-derived neurotrophic factor/neurotrophin-4, and neurotrophin-3 in ocular surface homeostasis and disease.

## 4. NGF

**Figure 3:**
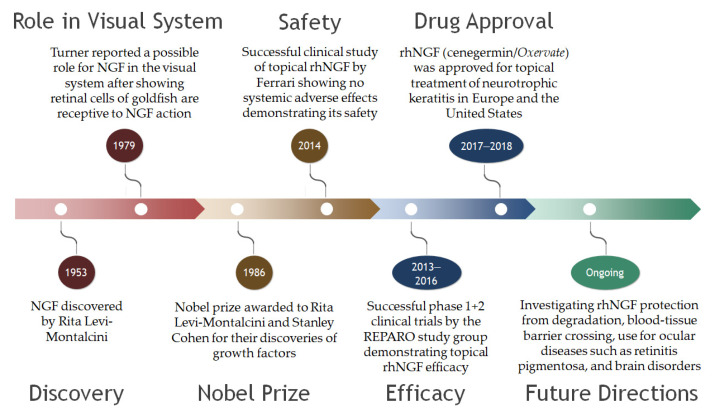
Timeline of the evolution of nerve growth factor from discovery to therapy. NGF, nerve growth factor; rhNGF, recombinant human nerve growth factor.

As mentioned above, NGF, the first neurotrophin discovered, has moved from bench to bedside (Figure 3). Neurotrophins play a significant role in homeostasis of the cornea, which is a densely innervated tissue of the body. NGF and the TrkA receptor expressed in normal aqueous humor and in tears modulate conjunctival and corneal cells [33]. Additionally, NGF plays a prevalent role in tear production and maintenance; it has been found in human and rat lacrimal gland tissues [25,34]. These observations indicate NGF is released continually at physiological levels. Tear flow is neurally regulated by autonomic and sensory innervation via local mediators [35]. NGF is one of these mediators, especially in dry eye disease, with a direct correlation between NGF levels in tears and level of corneal damage [36]. NGF is upregulated in corneal epithelial cells when they are under chronic hyperosmolar stress and can help reduce cell apoptosis and decrease NFkB activation levels [37,38,39]. In addition to its expression in the anterior segment, NGF is produced by the iris, ciliary body, lens, vitreous, choroid, and retina [40,41,42].

Promotion of wound healing and reinnervation by NGF in dry eye is now described.

### 4.1. Wound Healing

NGF and other growth factors released into the aqueous humor and tear film help regulate the growth and differentiation of corneal epithelial cells [43]. After corneal epithelial injury, there is increased expression of genes such as c-fos and c-jun that encode transcription factors in the trigeminal nuclei, which relay signals through the superior cervical ganglion, and hence, stimulate lacrimal gland cells to produce growth factors such as NGF [44]. Thus, increased NGF in rabbits with intraocular hypertension probably functions to provide increased endothelial support [45].

### 4.2. Corneal Reinnervation

Administration of NGF has been shown to accelerate cornea healing in rats [33,46], putatively via promoting limbal progenitor stem cell differentiation to maintain corneal epithelial cells [22] and regenerate nerves. Conversely, a reduction in NGF signaling has been reported to lead to reduced limbal stem cell extension [47]. These observations have motivated studies of NGF for treatment of ocular surface conditions such as dry eye, limbal stem cell deficiency associated with corneal surgery, neurotrophic keratopathy, and herpetic keratitis [47]. One such study demonstrated the efficacy of murine NGF on neurotrophic keratitis in humans, the use of which led to improvement in all patients and contributed to its development as a current drug [48]. After corneal refractive surgery in animal models consisting of rabbits, there was a detectable increase in NGF levels and faster recovery of the cornea after exogenous NGF administration [49,50]. In addition to enhancing limbal stem cell activity, NGF has reduced apoptosis of corneal epithelial cells, and increased corneal graft survival times by reducing the associated inflammatory state in rats [50]. For herpetic keratitis in rabbits, topical treatment with NGF helped reduce intensities of effects relative to acyclovir [50].

### 4.3. Dry Eye

The tear film creates an environment that maintains the epithelium of the cornea and the conjunctiva through mucin-mediated lubrication and barrier protection [51]. NGF concentration is increased in the tears of dry eye patients and is released by fibroblasts and epithelial cells of the cornea and the conjunctiva as well as local immune cells where it promotes cell survival [37,51,52,53]. Further, p75 and TrkA receptor levels generated by ocular surface epithelial cells, increase dry eye or injury, thus, promoting goblet cell mucin secretion [33,51]. In vitro, exogenous NGF has caused a dose-dependent increase in MUC5AC mRNA production, thus, inducing mucin secretion and stimulation of conjunctival epithelial cell differentiation in goblet cells [24,51]. In dogs with dry eye, NGF treatment increased tear production and goblet cell density [54]. An imbalance of neurotrophins in mice subjected to desiccating stress has been reported [55].

## 5. Clinical Trials Featuring NGF

In 2017, recombinant human NGF (rhNGF, cenegermin) was approved, in Europe, for topical treatment of moderate and severe neurotrophic keratopathy, and for all its stages, in the United States. Some features of studies leading to these approvals were as follows and as shown in Table 2. An escalating-dose phase I clinical study observed no systemic adverse effects after 5 days of topical rhNGF [56]. Then, a phase I clinical trial (REPARO study) explored the tolerability of 10 and 20 μg/mL rhNGF administered in eye drops 6 times/day for 8 weeks in 18 patients: only minor and transient ocular adverse effects were seen, and these did not require discontinuation of treatment [57]. The REPARO phase II study randomized 156 neurotrophic keratopathy patients from multiple European sites to either vehicle, 10, or 20 μg/mL rhNGF for 8 weeks [58]. In this study, 20% of the vehicle-treated patients and 55% and 58% of the 10 μg/mL and 20 μg/mL rhNGF-treated patients achieved corneal healing at 4 weeks. The percentages of responding patients grew to 43%, 75%, and 74%, respectively, of the total after 8 weeks of treatment, while maintaining the safety profile [58]. The NGF0214 trial randomized 48 neurotrophic keratitis patients from 11 sites in United States to 20 μg/mL cenegermin or vehicle eye drops/6 times daily for 8 weeks, then 24 weeks of follow-up [59]; 29% of the vehicle-treated patients and 70% of the cenegermin-treated patients achieved corneal healing after 8 weeks (defined as <0.5 mm of lesion staining), and if a more conservative definition of healing (no lesion or other residual staining) was applied, the difference between groups increased (17% vs. 65%, respectively).

In addition to these landmark studies, other reports have documented the efficacy of rhNGF in this disorder. In a prospective single-center study, the same dosage of topical cenegermin to 18 neurotrophic keratitis patients led to an increase in corneal sensitivity and subbasal nerve density from baseline, which could be interpreted as signs of corneal reinnervation [60]. In another prospective observational case series, a tear proteomic analysis of 15 patients with neurotrophic keratitis showed that topical cenegermin (20 µg/mL, 6 times/day) modulated inflammatory and neuroregenerative pathways in the ocular surface and increased corneal nerve fiber density after 4 and 8 weeks of treatment [61]. Further, cenegermin treatment led to increased best-corrected visual acuity and corneal nerve density in stage 1 neurotrophic keratitis; but, this was a retrospective study [62]. Regarding the applicability of rhNGF treatment for neurotrophic keratitis, real-life data from a German hospital agreed with clinical trial results by demonstrating complete epithelial defect closure and long-term improvement in visual acuity and corneal sensitivity [63]. There has also been observational evidence that positive effects of short-term rhNGF treatment for neurotrophic keratitis might have long-lasting effects in some patients [64,65,66]. Furthermore, reports or retrospective case studies of successful treatment of pediatric or congenital forms of neurotrophic keratitis have indicated that treatment with rhNGF showed efficacy [67,68,69,70,71].

Finally, indications for rhNGF other than neurotrophic keratitis are being explored. In a phase II open-label study, twice daily cenegermin (4 and 20 μg/mL) for 28 days led to improvement of dry eye symptoms and ocular surface damage [72]. There are at least two clinical trials currently underway that are analyzing the efficacy and safety of recombinant human NGF (cenegermin) for Sjögren’s dry eye disease (NCT05136170, NCT05133180). A phase 1 clinical study in open-angle glaucoma patients explored the safety and tolerability of short-term, high-dose rhNGF treatment (180 μg/mL cenegermin, 3 times daily, for 8 weeks) with a 24-week follow-up [73]. Although the nine-fold higher cenegermin concentration was well tolerated, no short-term neuroenhancement was observed. However, considering the strong preclinical evidence, the authors advocated for a human neuroprotection trial. Ocular topical delivery of rhNGF is also being explored for other ocular diseases such as retinitis pigmentosa [74] and even brain disorders [75].

## 6. Brain-Derived Neurotrophic Factor, Neurotrophin-4, and Neurotrophin-3

In contrast to NGF, investigations into effects of brain-derived neurotrophic factor, neurotrophin-4 (bind TrkB), and neurotrophin-3 (bind to TrkC) on the ocular surface are sparse, certainly less than those on the retina and on glaucoma [76,77]. Consequently, we summarize these neurotrophins together.

Brain-derived neurotrophic factor and neurotrophin-4 both bind to TrkB receptors, but they have different biological effects [78]. In synaptic activity, brain-derived neurotrophic factor and glucocorticoids share complementary activity [79] and it is possible that this may also happen in the ocular surface and its neurons. Brain-derived neurotrophic factor, neurotrophin-4, and neurotrophin-3 are present in the lacrimal gland, cornea, and conjunctiva [25]. Polymorphisms in the brain-derived neurotrophic factor gene were associated with dry eye in a study of 64 dry eye patients with 51 controls [80]. In cultured rat conjunctival cells, brain-derived neurotrophic factor, but not neurotrophin-3 and neurotrophin-4, stimulated secretion of glycoproteins without inducing proliferation [24]. In a mouse model of Sjögren syndrome that had corneal epithelial defects and reduced corneal mechanosensitivity and axon density, epithelial *Bdnf* transcripts were decreased in the corneal epithelium [81]. Further studies are needed to investigate the potential of brain-derived neurotrophic factor, neurotrophin-3, and neurotrophin-4 in cornea wound healing and reinnervation.

## 7. Neurotrophin Analogs

Neurotrophin analogs have been developed [82,83,84,85]. A small molecule, tavilermide (also known as **D3**) was designed to mimic *i* + 1, *i* + 2 residues of the 94, 95 turn in NGF. It proved to be a partial agonist of TrkA and did not bind TrkC or p75 [84]. Similar to NGF, tavilermide stimulated glycoprotein production in conjunctival cultures, increased phosphorylation of MAPK leading to activation in vitro, and improved corneal staining in an experimental dry eye model in mice [86]. Tavilermide has progressed to phase 3 clinical trials, underlining the potential of Trk agonists for treating dry eye. Toxic effects did not emerge in phase 2 [87]. The results from the phase 3 (study NCT03925727, clinicaltrials.gov) have not been published.

In addition to the obvious advantages (e.g., reduced cost of production, increased batch-to-batch reproducibility in manufacture, and increased stability in vivo), small molecule neurotrophin analogs have the advantage of only activating Trk receptors. Conversely, full-length proteins also activate the p75 receptor. This is important because the long-term effects of p75 stimulation by NGF analogs are unknown.

Consequently, we recently designed small novel Trk agonists, which we named **C1** (for selective binding to TrkC) and **pan** (for similar binding to all Trk receptors) [85]. **C1** and **pan** eye drops showed an inverse dose-dependent beneficial effect on cornea barrier function and goblet cell density in mice subjected to desiccating stress [85], a model of dry eye. **C1** and **pan** eye drops also increased transcripts of proteins involved in resolution of inflammation.

## 8. Conclusions

Neurotrophins are a closely related family of secreted proteins that promote neuron survival and participate in wound healing and epithelial health. Action of neurotrophins and their analogs via Trk receptors differentiate them from other compounds in the clinic, all of which have different modes of action. Recombinant human nerve growth factor (binds to TrkA) has been approved for clinical use for neurotrophic keratitis and it is under investigation for dry eye disease. However, little is known about the roles of TrkB and C in the cornea. Further research is needed to investigate the role of other Trk receptors in ocular surface repair.

## 9. Patents

Texas A&M has submitted a patent for small molecule compositions of matter, including **C1** and **pan** described in this publication.

## Figures and Tables

**Table 1:** List of abbreviations used in this review.

Akt	Protein kinase B
Erk	Extracellular signal regulated kinase
MAPK	Mitogen-activated protein kinase
NGF	Nerve growth factor
Raf	Rapidly accelerated fibrosarcoma
Ras	Rat sarcoma virus
rhNGF	Recombinant human nerve growth factor
Trk	Tropomyosin receptor kinase

**Table 2:** Clinical trials leading to FDA-approval of recombinant NGF.

NCT#/Reference	Phase	Sample (*N*, Sex, Age	Design	Results	Adverse Events (AE)
NCT01744704 [56]	I	74 healthy volunteers (24 F and 50 M, 40 ± 11 years)	Single, single ascending, or multiple ascending doses of rhNGF	No increase in serum NGF and no antidrug antibodies	Only mild, transient ocular AE (warm/pressure feeling, blurry vision, mild pain)
NCT01756456 [57]	I	18 Stage 2–3 NK patients (9 F and 9 M, range 24–86 years)	10 or 20 μg/mL rhNGF or vehicle, 6 drops/day for 8 weeks, then 48-week follow-up	Detectable serum NGF in 2 patients, no antidrug antibodies	Only mild ocular AE in 4 patients (29%)
NCT01756456 [58]	II	156 Stage 2–3 NK patients (95 F and 61 M, ~60 ± 14 years)	10 or 20 μg/mL rhNGF or vehicle, 6 drops/day for 8 weeks, then 48-week follow-up	55%, 58%, and 20% followed by 75%, 74%, and 43% corneal healing rates at 4 and 8 weeks, respectively	Only mild treatment-related AE in 25 patients (16%)
NCT02227147 [59]	II	48 Stage 2–3 NK patients (29 F and 19 M, 65 ± 14 years)	20 μg/mL rhNGF or vehicle, 6 drops daily for 8 weeks, then 24-week follow-up	70% vs. 29% corneal healing rate at 8 weeks, respectively	Mostly mild treatment-related AE in 18 patients (38%)

AE, adverse events; F, female; M, male: NF, neurotrophic keratitis

## Data Availability

Not applicable.
